# Ethical challenges in family caregivers of patients with advanced cancer – a qualitative study

**DOI:** 10.1186/s12904-020-00573-6

**Published:** 2020-05-18

**Authors:** Anneke Ullrich, Marianna Theochari, Corinna Bergelt, Gabriella Marx, Katharina Woellert, Carsten Bokemeyer, Karin Oechsle

**Affiliations:** 1 Palliative Care Unit, Department of Oncology, Hematology and BMT, University Medical Center Hamburg-Eppendorf, Hamburg, Germany; 2 Department of Medical Psychology, University Medical Center Hamburg-Eppendorf, Hamburg, Germany; 3 Department of Palliative Medicine, University Medical Center Goettingen, Goettingen, Germany; 4 Department of General Practice / Primary Care, University Medical Center Hamburg-Eppendorf, Hamburg, Germany; 5 Department of History and Ethics of Medicine, University Medical Center Eppendorf, Hamburg, Germany

**Keywords:** Caregivers, Cancer, Palliative care, End-of-life care, Ethics, Decision, Moral distress

## Abstract

**Background:**

Caring for patients with advanced or terminal diseases can confront family caregivers (FC) with ethical challenges. The present study aims at tracing paths connected to ethical challenges among FC of advanced cancer patients by exploring morally troubling situations and related burden, as well as strategies to handle the situation and experience of moral distress from the grieving FC’s perspective.

**Methods:**

Within a qualitative design, interviews with 12 grieving FC were conducted using a semi-structured interview guide. Data were analysed using grounded theory and abductive reasoning.

**Results:**

Core phenomena identified were two paths connected to ethical challenges among FC. Ethical challenges occurred in the context of difficult decision-making (Path 1) and in the context of lacking decision-making options when no decision was to be made by FC (Path 2). We found each path to be triggered by distinct sets of morally troubling situations that occurred during the patient’s disease trajectory. In the course of difficult decision-making (Path 1), detrimental external factors could add emotional stress, thus making the decision-making process burdensome. FC used various proactive strategies to overcome those detrimental factors and/or to make the decision. Decisions in conflict with FCs' own moral expectations and values led to moral distress, generating painful emotions. When no decision was to be made by FC (Path 2), FC felt powerless and overrun, which was associated with major emotionality in terms of anxiety and confusion. Either detrimental factors aggravated these feelings to paralyzing shock, or internal resources enabled FC to accept the situation. While acceptance prevented moral distress, paralyzing shock often caused a sense of not meeting their their own moral expectations and values, resulting in moral distress. In both paths, factors were identified that helped FC finding closure and prevented moral residue. Nevertheless, some FC experienced residual moral distress months after the morally troubling situation had occurred.

**Conclusion:**

Findings provide first information towards understanding paths leading to ethical challenges in FC and can help clinicians to minimize associated emotional burden and moral distress.

## Background

Family caregivers (FC), e.g. partners, relatives and friends who care for the patient, are a key resource for the well-being of terminally ill patients, but also by their assistance and active involvement in treatment decisions and care planning [1]. In the trajectory of an incurable, progressive disease, patients and their FC are confronted with many difficult decisions influencing further care planning and quality of life, such as decisions about life-prolonging treatment, medically assisted nutrition and hydration, transitions in care (e.g. seeking emergency care), or the place of care and death [2–6]. In palliative and end-of-life care, many of these decisions necessitate difficult conversations, need to be taken ad hoc, are irreversible, and are of existential meaning for the patient and his or her family. Since FC are usually most acquainted with the patient’s values and cater most about his or her best interests [2], they often participate in medical decision-making processes [2, 7, 8]. Prior studies demonstrat that many patients prefer family involvement in decisions [9] and that FC voice their wish to be involved, too [10]. Between 49 and 84% of cancer patients and 54 and 59% of FC prefer to involve FC in the decision-making process [8]. While recognizing potential problems such as FCs’ dominant behavior, oncologists also appreciate family involvement in decision-making [11, 12].

However, studies suggest that at least one-third of FC face emotional pressure and decisional burden associated with doubt and regret months or even years afterwards, which could lead to depression and stronger grief [8, 13]. High caregiver burden has been reported specifically in case of substitute decision-making responsibilities, uncertainty about the patient’s wishes and values, and conflicting wishes regarding the place of death [13–15]. In the context of palliative and end-of-life care, FC may encounter manifold morally distressing problems or ethical dilemmas. These, among others, include withholding/withdrawing of treatment, nutrition and hydration, resuscitation orders, palliative sedation, and truthful communication [16, 17].

Stress due to ethical dilemmas has often been referred to as “moral distress”, which was first described by Jameton [18] and has become an increasingly prevalent topic of discussion in healthcare [19]. It describes the burden of a person when dealing with ethical dilemmas because he or she could not act according to their own values due to internal or external constraints [18, 20]. Moral distress has been found to lead to feelings of depression, helplessness, exhaustion, frustration, guilt, and self-accusation [21, 22]. Moral distress can either linger after the causal event, or can even grow with time, which has been called moral residue [22]. Despite its original use for experiences of healthcare providers [18], lately, some authors have argued that the concept of moral distress should be broadened [23]. It can be assumed that morally troubling situations and dilemmas may lead to moral distress among FC of terminally ill patients. However, specific prerequisites of the caregiver role, such as direct personal involvement (vs. professional near-distance structure in healthcare providers) and a lower background of knowledge and experience (vs. professional routine in healthcare providers) may influence the nature of moral distress.

Knowledge about how ethical challenges occuring from morally troubling situations among FC could help to identify strategies on how to prevent and reduce related burden and eventually moral distress. However, research has mainly examined the perspective of patients themselves, healthcare providers and ethicists on ethical conflicts and dilemmas in the care of cancer patients [24]. Ethical challenges that FC of advanced cancer patients may experience across the patient’s disease trajectory have been rarely investigated. Thus, this study aimed to gain insights into paths connected to ethical challenges by exploring morally troubling situations as well as related burden, strategies to handle the situation, and the experience of moral distress from the grieving FC’s perspective.

## Methods

### Study design, setting and participants

This qualitative study was conducted using the grounded theory of Glaser and Strauss [25], further developed by Strauss and Corbin [26]. The study draws on interviews with grieving FC of advanced cancer patients, who had received specialist inpatient palliative care at the palliative care ward of the University Medical Center Eppendorf, Hamburg, Germany. All FC approached for interviewing had participated in a larger quantitative study on FCs' psychosocial burden [27], which did not examine ethical challenges or moral distress. FC, irrespective of being a family member or friend, were eligible if they were aged > 18 years, had been indicated as the primary caregiver by the deceased patient, and were fluent in the German language.

FC included in the study share a past life experience in common (life history homogeneity [28]). Our rationale was to capture the variation across these FC to provide a scope for the development of cross-case commonalities and diversities regarding ethical challenges [28, 29]. Thus, a purposive sampling strategy was used to choose participants for intensive interviewing regarding groupings of age, kind of relationship to the patient, and time since the patient’s incurable diagnosis.

The first authors, A.U. and M.T., none of them involved in the care provided to the FC or the deceased patients, recruited eligible FC by phone. Recruitment of FC was continued until no new codes derived from the interviews and data saturation was reached [30]. According to proposed principles for specification of data saturation [31], we a priori specified a) an initial analysis sample for the first round of analysis (4 interviews), and b) a number of further interviews that had to be conducted after the point at which no new codes were identified (2 interviews).

The ethics committee of the General Medical Council of Hamburg, Germany, approved the study protocol (reference number PV5122). FC invited for an interview had consented to be contacted for a later interview request during the preceding quantitative study [27], and for all participating FC written informed consent prior to the interview was mandatory.

### Data collection

Adopting a narrative approach, data were collected through interviews basing on a semi-structured interview guide. For reasons of quality assurance, we used a framework for the systematic development of the interview guide [32]. The multi-professional research team designed the guide drawing on existing evidence and clinical experience. We conducted a pilot interview in order to test and redefine our guide. Since we undertook only insignificant adjustments to the interview guide, the pilot interview was included in the final data analyses. Table 1 gives an overview of central items of the interview guide.

**Table 1:** Semi-structured interview guide

**Introduction:** Presentation of one’s own person. Recognition of the special situation through the loss of the patient and appreciation of the participation in the interview. Explanation of the goals of the study and interviewing. **Opening question (narration):** Can you describe specific decisions or situations that were difficult for you for ethical reasons? Were there decisions or situations where you wondered if you were or another person were doing the right thing? Please consider the time from the patient’s incurable diagnosis until death. You can take as much time as you want for telling me about your experiences. **Exmanent questions (when aspects were not mentioned during the narration, or to deepen the narration):** When you think of the decision or situation you have described … *Exploring morally troubling decisions or situations:* When did you experience this decision or situation in the course of the patient’s disease? What happened? Who was involved? *Exploring emotional burden:* How burdensome was it for you to be confronted with the decision or situation? Can you describe your feelings during that decision or situation? What stressors did you face? *Exploring resources:* Can you tell me how you dealt with the decision or situation? What did help you? What were sources of strength? *Exploring needs when dealing with the decision or situation:* What were your needs for information, advice and support? Were the needs met and if so, by whom / through what assistance? In which cases have needs / wishes possibly not been sufficiently taken into account? *Exploring unresolved decisions or situations:* Do you remember decisions or situations that you did not think could be solved or not satisfactorily? What were the reasons? *Exploring moral distress:* How much did the decision or the action you took / did not take correspond with your own moral expectations or values? If it did not, how did you feel at this time? What did help you to deal with these feelings? How do you currently feel (at peace, still bothering)? **Closing the interview** Do you have any questions or comments? Are there aspects that are important to you that have not yet been discussed in the interview? How do you feel now? How did you experience the interview?	

Starting with the opening question, the interviewees were encouraged to narrate about morally troubling situations or questions they experienced during the patient’s disease trajectory, starting with the patient’s diagnosis of incurable cancer. Exmanent open questions were translated to immanent questions, using the language of the interviewee, to expand the narrative on how they felt about eventual decisions at that time, in which way they felt burdened by the situation, which resources and strategies they used, and how they judged these situations in retrospect. Through that semi-structured approach, we made sure to cover specific relevant topics, but at the same time leave room for the interviewees individual verbal expressions and improvised use of follow-up questions [32].

Two female interviewers, A.U. (sociologist and psychooncologist, highly trained in interviewing) and M.T. (MD, medical doctorate candidate, trained by A.U.), conducted the interviews between October 2017 and April 2018. At the beginning of each interview, the interviewers introduced themselves according to their credentials and their research focus on family caregiving in the context of palliative and end-of-life care. Further, the aims and the reasons for doing this study were explained. Interviews were carried out one-to-one, except for the pilot-interview, which was carried out by A.U. at the presence of M.T. to convey interviewing skills in a real life setting. All interviews were face-to-face interviews, and took place at the premises of the University Medical Center. To avoid selection bias regarding the age of participants, interviewees could choose to be interviewed during or after working hours. Further, we anticipated that some interviewees could feel uncomfortable when revisiting the palliative care ward. Thus, interviews were either conducted at the palliative care ward or a neutral room as preferred by FC. During the interview, the interviewers ensured that collecting data was always secondary to FCs’ well-being. Questions were phrased sensitively in accordance with techniques for interviewing vulnerable people in palliative care settings [33, 34], allowing FC time for reflection. Upon request, the interviewees received the audio file of the interview.

After the interview, interviewers made ad hoc field notes on key topics and hypotheses emerging during the interview, as well as a rough outline of the main components of the narrated situations and the action strategies used to handle them. These notes were later used as an aid in the analysis of the data. Transcripts were not returned to the interviewees for corrections or feedback.

### Recruitment process and family caregiver characteristics

We completed 12 in-depth interviews with FC. In the recruitment process, a further eight FC who had been contacted had declined interview participation. Reasons were feeling incapable of attending a face-to-face interview, either because of high grief-related emotional burden (*n* = 3), having moved far away (n = 3) or facing own serious health issues (*n* = 2).

Eight of 12 interviewees were female and age varied between 36 and 74 (mean 55.6 ± 14.5 years). Eight FC were a partner to the deceased, the remaining FC were children (n = 2), a close friend or a parent, respectively. Elapsed time since the patient’s death ranged from five to 9 months, and the final place of death had been a palliative care ward in seven cases (Table 2). Interviews on average lasted 75.6 min (range 50–102).

**Table 2:** Demographic details of interviewed family caregivers (*N* = 12)

ID	FC‘s gender	Kind of relationship. FC was …	Years FC knew patient in years	FC was appointed as substitute decision-maker^1^	Time between diagnosis and admittance to palliative care ward	Time since patient’s death in months	Final place of patient’s death
1	Female	Partner	35	Yes	2–5 years	6	Palliative care ward
2	Female	Partner	15	Yes	3–6 months	6	Inpatient hospice
3	Female	Partner	30	Yes	2–5 years	5	Inpatient hospice
4	Female	Partner	31	Yes	1–2 years	7	Palliative care ward
5	Female	Parent	45	No	5–10 years	5	At home with specialist palliative care
6	Male	Child	36	Yes	1–2 years	6	Nursing home
7	Male	Child	44	No	< 3 months	6	Palliative care ward
8	Female	Partner	16	Yes	> 10 years	8	Palliative care ward
9	Male	Partner	55	No	1–2 years	6	Palliative care ward
10	Female	Partner	30	Yes	1–2 years	6	Palliative care ward
11	Male	Close friend	20	Yes	6–12 months	6	Inpatient hospice
12	Female	Partner	14	No	2–5 years	9	Palliative care ward

^1^ During the disease trajectory, the patient had appointed the FC to act as substitute decision-maker in terms of personal (including health) matters. Thus, the FC was permitted under the law to make decisions on behalf of the patient regarding medical decisions, if the patient lacked decision-making capacity

Abbreviations:*FC*Family caregivers

### Data analysis

The interviews were audio-recorded and transcribed verbatim with all person-related information being pseudonymized. Neither interview transcripts nor findings of the study were returned to the interviewees for corrections or feedback. Transcripts were analyzed by M.T., supervised by A.U., and discussed within the multi-professional research team on a regular basis (every 2 to 4 weeks troughout the analysis period). The software MAXQDA facilitated data management and coding.

Transcripts and field notes were analyzed using grounded theory and abductive reasoning, including all steps of open, axial and selective coding [25, 26]. With grounded theory techniques, our purpose was to generate a theory of ethical challenges from the FC’s perspective by identifying salient patterns and distinguishing the relationships among them. However, we were also interested in understanding the temporality and plot of such ethical challenges. Thus, we integrated elements of narrative techniques in the data analysis process, to consider the dimension of sequencing of core narratives within an interview [35].

To identify patterns and how they relate, codes were inductively developed using the coding paradigm by Strauss and Corbin [26] to structure the analysis process. The coding paradigm refers to causal conditions leading to a core phenomenon, the context of the phenomenon, intervening conditions, action strategies to handle the situation and consequences of the action or the core phenomenon. Open and axial coding identified concepts, which in an iterative constant comparison approach resulted in categories and sub-categories. Axial and selective coding was performed in an iterative process until no new codes emerged and core phenomena had become evident [26]. In the final step of analysis, we linked categories that resulted from the grounded theory analysis into the FC’s story to include temporality. We examined the FC’s experience through temporal concepts by identifying aspects that moved the narrated story forward. That way a theoretical framework was developed for understanding the paths leading to ethical challenges among FC.

We used the Consolidated Criteria for Reporting Qualitative Studies (COREQ) framework to report on the design, analysis, and results of our study [36].

## Results

### Categories developed

The core phenomena identified were two paths connected to ethical challenges among FC. Ethical challenges occurred in the context of difficult decision-making (*Path 1*) and in the context of lacking decision-making options when no decision was to be made by FC (*Path 2*).

We found these paths to be triggered by distinct types of morally troubling situations that occurred during the patient’s disease trajectory, from the time of the incurable cancer diagnosis until the patient’s death. These situations are following referred to as predisposing factors. FC did not necessarily reflect the ethical dimension of these situations in their narrations. However, all of them included ethical issues and dilemmas as described by Beauchamp and Childress [37] or as “meaningful experiences and situations in general, which concern the fundamental questions of human life” [38]. The latter posed, for example, the confrontation with the fragility of human life, the experience of relational autonomy, and concerns for others.

**Fig. 1:**
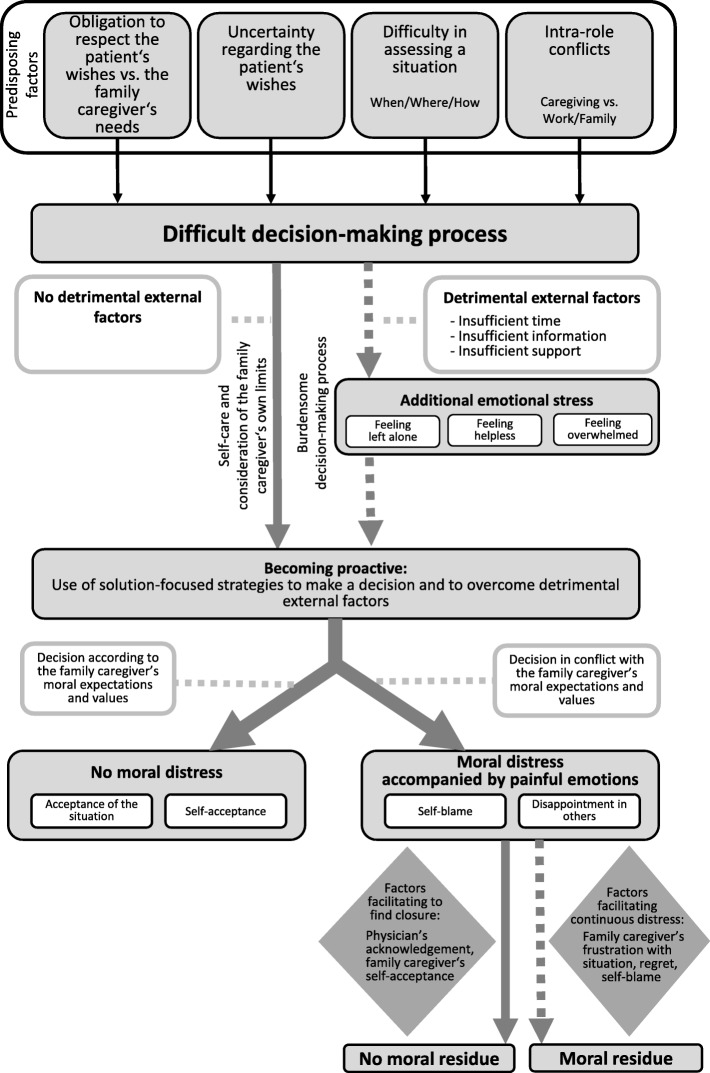
Path 1: Ethical challenges in the context of a difficult decision-making process

In the course of a difficult decision-making process (*Path 1*; see Fig. 1), detrimental external factors could add emotional stress, thus making the decision-making process burdensome. Proactive strategies were used to overcome these obstacles or to make the decision. In some FC psychological response was moral distress, which occurred when FC could not act or decide according to their own moral expectations and values, such as respect for the patient’s autonomy.

**Fig. 2:**
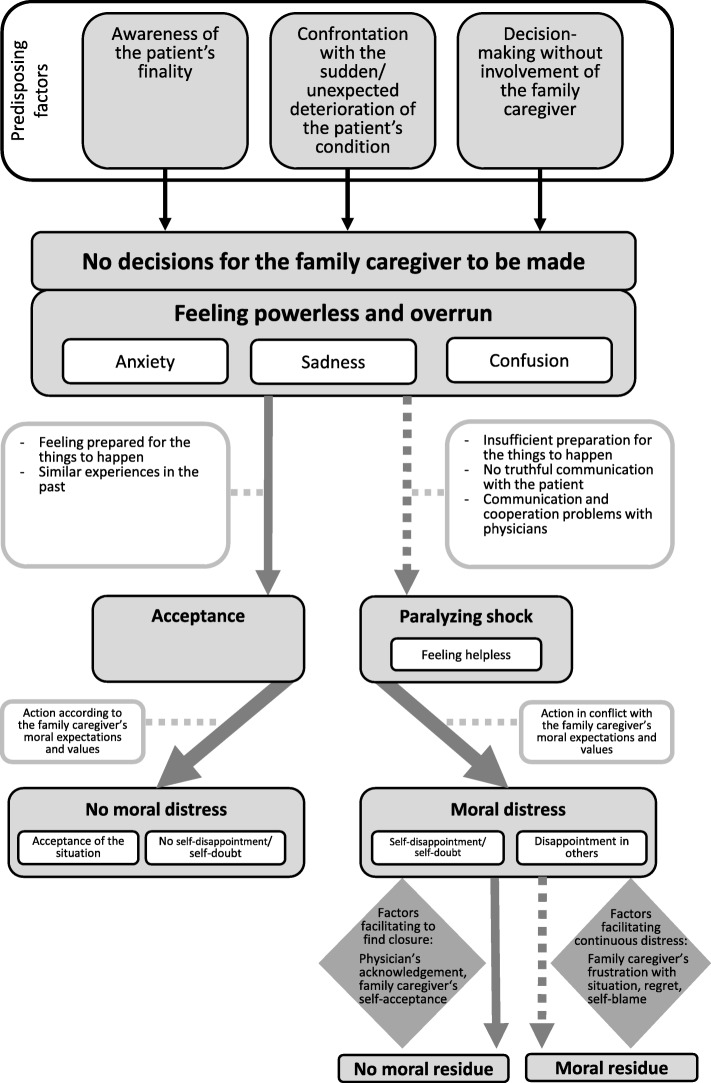
Path 2: Ethical challenges when no decision was to be made by caregivers

When there was no decision for the FC to be made (*Path 2*; see Fig. 2), analyes showed that FC felt powerless and being overrun, which was accompanied by manifold painful emotions. Either detrimental factors aggravated these feelings to paralyzing shock, or internal resources enabled FC to accept the situation. While acceptance prevented moral distress, paralyzing shock often caused a sense of not meeting their own moral expectations and values, such as honesty, resulting in moral distress.

In both paths, retrospectively, factors allayed the experience of moral distress. These included external resources, often related to physicians’ behavior, as well as FC’s internal resources like self-acceptance. However, some FC experienced moral residue months after the morally troubling situation had occurred.

Most FC described more than one morally troubling situation (predisposing factors) during the interview. FC narrated how they dealth with each experienced situation and which factors led to emotional burden and/or moral distress. This way details on the commonalities and the diversities of FCs’ experiences emerged, and the two paths connected to ethical challenges became evident. In many cases, both paths were identified in one FC according to the morally troubling situations he or she described.

### Path 1: ethical challenges in the context of a difficult decision-making process

A visualization of *Path 1* is displayed in Fig. 1. It shows key elements linked to emotional burden and moral distress in the course of a difficult decision-making process, which in detail are as follows:

#### Situations triggering a difficult decision-making process

During the disease trajectory of the patient, a set of four types of situations triggered a difficult decision-making process among FC. The following four sub-categories of such predisposing factors were demonstrated:

FC experienced decision-making as challenging,

1. if their own needs did not correspond with the patient’s wishes. FC reported that although they felt obliged to respect the patient’s wishes, they knew there was no alternative. An often mentioned example was that referral to a palliative care ward or an inpatient hospice was initiated due to the FC’s physical or mental burden, though the patient’s expressed his or her wish for home care (*Obligation to respect the patient’s wishes* vs. *FC’s needs*).
  *“Well, that was tough, when he was admitted to the palliative care ward because I think he somehow knew that he wouldn’t go back home. That is why he resisted coming in. Nevertheless, it was clearly no longer possible at home. That was a difficult decision for me.”*
2. if FC were not sure whether they perceived and interpreted the patient’s needs in the right manner, e.g. in cases of patient’s diminished communicative and cognitive abilities (*Uncertainty regarding the patient’s wishes*);
  *“We thought my mother wanted the children to keep her in their minds, as she was. I wanted to respect her at that moment. I think it was bad that my children did not say goodbye. I think she really missed that at the end.”*
3. if FC felt unprepared and uninformed for the experienced consequences of their decisions and the symptoms that will manifest, thus feeling incapable to assess a situation. As an example, they named the patient’s dying process and their difficulty to assess the right time (“when”) and the right place (“where”) for it, especially if they felt that they had been insufficiently informed about the course of the dying process (“how”) (*Difficulty in assessing a situation*);
  *“For me, it was always clear that from a point in time, when there’s no way back and he suffers and has severe impairments, that he would not want to prolong it...But then, it is difficult to find this exact point in time.”*

(4) if FC faced an intra-role conflict and felt torn because of the conflicting expectations between their different roles (caregiving, work, needs of family and friends) *(Intra-role conflicts)*.
  *“That was a tough time. This ‘sandwich position’, when my own family also claimed their needs … and work!”*

#### Factors making a difficult decision process burdensome and use of action strategies to handle the situation

Although FC did not necessarily reflect a difficult decision-making process to be ethically challenging, they recognized a high emotional burden once external detrimental external factors occurred during decision-making. Relevant detrimental factors were insufficient time, information, or support, which are listed in Table 3 in detail. FC narrated that they felt helpless, overwhelmed, and left alone if burdened due to external obstacles, which aggravated the decision-making process from the FC perspective. On the other hand, if there were no external obstacles that would have additionally stressed them from their perspective, they deliberately acted in a self-protective manner, taking into account their own limits and putting self-care as a high priority. In both cases, this led them to develop different personal action strategies to overcome these obstacles or to make the decision. They took initiative by asking for more time to make the decision, or they looked for help, support and information from the social environment, from befriended physicians, or from other healthcare providers (e.g. nurses, psychologists).

**Table 3:** Detrimental external factors that led to emotional burden among family caregivers in the context of a difficult decision-making process *(Path 1)*

External factors that led to emotional burden	
**Insufficient Time**	• To make the decision • To confront the situation • To reevaluate the situation • To prepare for what is to come
**Insufficient Information**	• About the consequences of the decision for their loved ones and their suffering • About the length of time • Discrepancy: “For a long time nothing is said, and then come sudden announcements”
**Insufficient Support**	• From the social environment • From the family • From physicians / specialist outpatient palliative care teams • From the patient

#### Experiencing moral distress and factors that facilitate finding closure

After the use of proactive strategies, the analysis showed FC to be in high distress if they could not act according to their moral expectations or values. In particular, values related to “good” caregiving, patient autonomy and patient will, integrity and honesty were violated. For example, FC could not execute their perceived obligation for parent care due to workplace responsibilities. Thus, they felt like knowing the right thing to do (value: filial responsibility as an expression of love or debt of gratitude), but were constrained to act accordingly. Other FC witnessed disrespectful treatment of the patient by other family members or healthcare providers, compromising patient autonomy. Although recognizing the morally appropriate action (value: primacy of patient autonomy), constraints like lack of courage or experience prevented them from preserving the values at stake. Distress resulting from the violation of the FC’s moral expectations and values, defined as moral distress, included painful feelings of self-blame, self-doubt, and self-disappointment, or disappointment in others. Some FC referred to such experiences during the disease trajectory and indicated that they could find closure in retrospect. However, some FC identified their residual frustration and self-blame a long time after the challenging situation had occurred. We found factors that facilitated to find closure and prevent moral residue: The acknowledgment of the caregiver role and the validation of the FC’s emotions by physicians (external resources), as well as the FC's self-acceptance and their not having regrets about the loved one‘s death (internal resources). Some FC initiated a discussion with the physicians after the death of the patient in their need of clarifying the ownership of presumed mistakes regarding the patient’s treatment on behalf of the physicians. An empathetic, reflecting and understanding approach from the physicians helped to prevent moral residue.

### Path 2: ethical challenges when no decision was to be made by caregivers

Figure 2 visualizes *Path 2* and shows key elements linked to FCs’ emotional burden and moral distress in the course of lacking decision-making options, which in detail are as follows:

#### Situations when there were no decisions to be made

During the patient’s disease trajectory, various morally troubling situations were identified that led to a perceived lack of decision-making options. The following three sub-categories of such predisposing factors were found:

FC experienced decision making as challenging,

1. if they gained full awareness of the finality and the definitiveness of the situation and realized that their loved ones would not improve, accompanied by the feeling that they could no longer “do anything”. Examples included infections in patients not responding to antibiotic treatment at the end of life (*Awareness of the patient’s finality*);
  *“It was a difficult time for me. This awareness that something is coming to an end. You don’t really believe that... [begins to cry] You always think: “He can do it”.*
2. if they were confronted with unexpected symptoms, the unexpected progression or course of the disease and shocked by how fast the disease could develop. Descriptions of such situations were found across all FC (*Confrontation with the unexpected deterioration of the patient’s condition*);
  *“That was so fast … we all did not know...It was like a Tsunami for us … We relied on the doctors, on their statements [sighs] “.*
3. if FC were presented with a fait accompli because the patient made a decision without taking their opinion into account and they were completely taken by surprise. A frequently given example was that the patient stopped a vital medical treatment without telling the FC *(Decision-making without involvement of the FC)****.***
  *“That was something that made me very sad, that he had not previously said: “What do you think?“, or if he had said to me: “Well, this is so exhausting... What do you think about stopping therapy?“ I don’t know how I would have reacted, but … he decided that for himself. I only had to accept that. I often thought: “You are not even included...you’re not that important.”*

#### Feeling powerless and factors leading to paralyzing shock or acceptance

Due to the perception of lacking decision-making options, feeling powerless and overrun was particularly challenging for FC and was accompanied by deep feelings of anxiety, sadness, and confusion. However, FC showed two different ways of reacting to those feelings depending on external and internal factors: If they felt prepared for the outcome – upon sufficient communication and information shared by physicians or because they had already made similar experiences in the past – they could make better use of their own resources and consequently accept the situation. Conversely, detrimental factors led to paralyzing shock and helplessness among affected FC. Such factors were insufficient preparedness for the expected course of the disease and death, no truthful communication with the patient, or communication and cooperation problems with physicians (Table 4).

**Table 4:** Detrimental factors that led family caregivers to feel shocked in the context of lack of decision-making options *(Path 2)*

Factors that led to paralyzing shock	
**Insufficient preparation**	• „Ups and Downs “in the disease trajectory: hope and disappointment • Physicians don’t take family caregivers through the individual steps • Not feeling prepared for changing goal of care, occurring symptoms, deteriorating health status, dying and death
**No truthful communication with the patient**	• Patient autonomy over caregiver’s needs • Patient withholding information • Patient not wanting to talk about the disease, death and dying • No agreement with the patient about medical decisions • Reciprocal protection
**Communication and cooperation problems with physicians**	• Lack of empathy • Unclear statements • False hope • Indecision and inconsistency

#### Experiencing moral distress and factors that facilitate finding closure

In the acceptance scenario, a key element helping to avoid moral distress was FCs' feeling that they could not have done anything better and reported to be at peace with themselves and their moral obligations. In contrast, shock and helplessness led to FCs' feeling that they could not meet their own expectations regarding supporting and caring for their loved ones. This condition showed to be very stressful and often led to moral distress due to violation of the FC’s moral expectations and values. For example, FC narrated that they felt morally responsible to act selflessly or to be continually present in hopes of fostering the patient’s well-being when death approaches. However, due to perceived constraints like helplessness, some claimed that they did not do enough for the patient (value: primacy of the patient’s well-being). To oblige the patient’s preference, some FC did not talk truthfully to the patient about their own concerns or topics that were relevant to them, such as dying and death. As a consequence, they felt that they acted contrary to their personal values (value: honesty in a close relationship).

In analogy to *Path 1*, factors facilitating to find closure and preventing moral residue comprised external and internatal resources: FC pointed out the importance of physicians’ acknowledgment of the caregiver role and clarification of the ownership of presumed mistakes (external resources) as well as self-acceptance (internal resources). Otherwise, they reported ongoing feelings of frustration and blaming themselves for wrongdoing, and as result being incapable of finding closure and experiencing moral residue.

## Discussion

This qualitative study delves into paths connected to ethical challenges among FC during the advanced cancer trajectory and how FC could be supported to prevent or reduce the related emotional burden or moral distress. We found two paths arising from different morally troubling situations and manifesting themselves differently depending on various factors. Yet, paths appear to coincide when it comes to their impact on FC moral distress and moral residue.

We found distinct types of morally troubling situations that either triggered a difficult decision-making process (*Path 1*) or lacking decision-making options (*Path 2*) from the FC perspective. Regarding *Path 1*, FC described situations characterized by uncertainty regarding the patient’s preferences or diverging patients’ and FCs’ needs. The impact of such challenges is consistent with studies demonstrating decisional burden of FC in cases of uncertainty about patient’s wishes and conflicting needs, e.g. regarding the place of care and death [13, 15]. For example, a study on FC who made a surrogate decision about the place of end-of-life care showed that FC reported significantly more burden when the decision was not concordant with the patient’s wishes [15]. Another type of morally troubling situations was dilemmas arsing from conflicts between caregiving and other commitments like family or work. Consistent with our finding, challenges related to competing roles of FC have been eludicated in the discussion about ethical dimensions of filial caregiving [39]. With respect to *Path 2*, FC described situations which confronted them harshly with the finality of the patient’s life or sudden deterioration of his or her health status. FC felt powerless and overrun, since no decisions or options regarding cancer treatment or life-prolonging treatment were left. Additionally, FC experienced that patients chose to not involve FC in decision-making, e.g. treatment choices. Altogether, morally troubling situations as described by FC often included aspects concerning relational autonomy [40], e.g. in terms of inter-relatedness of decisions that have to be taken, and concerns for others, e.g. in terms of achieving consensus.

In the course of both paths connected to ethical challenges, FC described detrimental external factors that, under the given circumstances, added emotional burden. Our findings showed that sufficient time, information and support from the social environment and from the physicians early on could prevent elevated burden. The necessity of providing FC with enough time and emotional support during decision-making processes has been highlighted in previous studies [15]. Relatedly, FC could be better prepared for the upcoming events through adequate form and content of caregiver-physician communication, and eventually deal with or even avoid moral distress. Prior works have described the importance of good communication skills of healthcare providers to sufficiently prepare FC for substitute decision-making [15] and discussions around end-of-life care decisions [41]. Strategies to improve FCs’ preparedness include the use of online tools for advance care planning [42] or palliative care-led family meetings [43]. Ethical guidance for healthcare providers to optimize relationships with FC that also address communication in palliative care and end-of-life scenarios are available [7]. Healthcare providers can offer ethical support, e.g. by assisting FC to recognize and discuss the ethical dimension of their experiences. FC should be prepared that probably difficult decisions will come up in the disease trajectory, which may be perceived as morally troubling. Professionals may take the therapeutic opportunity to communicate that many FC share such experiences under these circumstances, reminding them that people are “moral agents” [19]. FC can be informed about available sources of support in dealing with morally troubling situations and emotional discomfort.

FC described being in high distress when they could not act according to their moral expectations and values due to perceived external or internal constraints. They felt disappointed in themselves or in the physicians, and doubted about their decisions or actions. This distress as a consequence of the violation of FCs' moral expectations and values is referred to as moral distress [18], which involves a crisis of conscience [20]. It differs from emotional distress [18, 22] and has been described as a highly burdensome and negative experience in FC [44]. Yet, moral distress seems to be an under-recognized phenomenon in the vulnerable situation of family caregiving [45]. While emotional burden may be addressed by counselling and access to psychological support for FC, presumed moral distress or ethical issues might request the inclusion of clinical ethicists. Research shows that different forms of clinical ethics support exist, which give advice and recommendation to healthcare providers, patients and their families [46, 47]. One way to support FC in dealing with ethical challenges has been through ethical consultations. A study suggested that consultations helped FC in various ways including increased clarity on the ethical problem, facilitation of the decision-making process, and consolation [47]. In order to optimize supportive care for FC in potentially ethically challenging situations, offers of support should stay flexible and adaptable to meet FCs' needs, allowing for the integration of ethical counselling. Further, it has to be considered that FC involved in decision-making or acting as substitute decision-makers operate in interpersonal relationship networks with social and moral obligations. Complex agendas, needs and interpersonal dynamics in the family system may shape the experiences and decision-making of FC in palliative and end-of-life care [48, 49]. To better understand such a complex phenomenon as ethical challenges in FC of terminally ill patients, a systemic approach that accounts for the interactions, mutual influences, hierarchies and boundaries of family systems may be helpful in psychological and ethical counselling.

Moreover, FC that experienced moral distress noted that physicians’ empathetic approach and acknowledgment of the caregiver role in addition to the validation of their emotions helped them find closure. Physicians’ ownership and acknowledgment of presumed mistakes that may have happened during the patient’s disease trajectory, could potentially reduce the FCs’ feelings of disappointment and frustration and prevent moral residue, which is congruent with the results of a previous study [7]. FC also described that self-acceptance and having no regrets about the loved one’s death as pivotal regarding the prevention of moral residue. Empirical evidence confirms the considerable role of regret among FC during palliative and end-of-life care or bereavement. Types of regret reported in the literature include something FC were not able to do for the deceased, the decision to admit a patient to a palliative care ward, not insisting for better care, and not having talked about death [50–53]. The role of healthcare providers as well as clinical ethicists could be to facilitate awareness and communication about end-of-life decisions, and to support FCs' reflections on ethical issues to reduce ambiguity and regret.

### Strengths and limitations

Our study has some strengths and limitations. By interviewing grieving FC of terminally ill patients, we investigated the unique experiences from the subjective perspective of this person subgroup itself, which is a major strength. Our endeavor of validating our findings included analysis and discussion of data by a multi-professional research team. Using the grounded theory approach and abductive reasoning, we were able to generate an in-depth understanding and explanations for paths connected to ethical challenges in FC. Regarding the limitations of our study, caution is required regarding the generalizablility. Our results cannot be generalized to FC of patients with other chronic diseases than cancer (e.g. dementia, organ failure), since disease trajectories differ significantly [54]. Nevertheless, we are positive, that our study provides important clinical implications that are transferable to non-cancer settings. Furthermore, the missing perspectives of the grieving FCs' who declined interview participation due to own serious health issues or grief-related emotional burden may have biased our findings, and we may not have reached theoretical saturation for the group of FC struggling with negative physical or psychological health conditions. The retrospective reinterpretation of morally troubling situations possibly affects the description of the experienced. However, after-death interviews are an important tool to study the situation of FC in the context of advanced cancer [55, 56].

## Conclusion

Ethical challenges add complexity to the caregiving experience of FC in palliative and end-of-life care. Findings can be used as guidance for healthcare providers to early detect morally troubling situations, as they are a potential source of ethical challenges in family caregiving for a terminally ill patient. Although healthcare providers cannot prevent FC from experiencing morally troubling situations, they should be aware of ethical issues that may arise. Providers can, for example, assist FC by helping to identify ethical issues, by enabling FC to reflect and verbalize burdening aspects and emotionality, and by clarifying FCs’ (lacking) resources and coping skills. We identified specific detrimental factors, which caused additional emotional burden and moral distress after a morally situation had occured. Knowledge on these factors may increase healthcare providers’ sensitivity related to communication styles, information giving and emotional support of FC who experience morally troubling situation. Our findings might assist in developing practice guidelines and interventions for the target group of FC caring for terminally ill cancer patients as well as training for healthcare providers. Training may strengthen providers’ skills to proactively deal with affected FC, e.g. how to detect and manage ethical challenges in FC, and how to maintain an effective collaboration with FC under these circumstances. Future research is needed to specifically explore factors that result in moral residue among FC in palliative and end-of-life care, and ways to prevent it.

## Data Availability

The authors have full control over the primary data. The data analyzed in this study are housed at the Palliative Care Unit, Department of Oncology, Hematology, and BMT, University Medical Center Hamburg-Eppendorf, Martinistrasse 52, 20246 Hamburg, Germany. As per the ethical committee approval, this dataset is subject to ethical restrictions, and informed written consent of study participants does not include the publication of raw data in terms of interview manuscripts.

## Abbreviation

FC: Family caregivers
